# Telephone Counseling for Children Recovering from Tonsil Surgery—A Randomized Controlled Feasibility Study

**DOI:** 10.3390/healthcare12181862

**Published:** 2024-09-16

**Authors:** Helena Rosén, Kajsa Landgren, Eva Olofsson, Eva Drevenhorn, Gunnhildur Gudnadottir, Rebecca Gagnemo Persson

**Affiliations:** 1 Department of Health Sciences, Faculty of Medicine, Lund University, P.O. Box 157, SE-221 00 Lund, Sweden; kajsa.landgren@med.lu.se (K.L.); eva.drevenhorn@gmail.com (E.D.); rebecca.gagnemo_persson@med.lu.se (R.G.P.); 2 Department of Paediatric and Adolescent Medical Care, Skaraborg Hospital, 54185 Skövde, Sweden; 3 Department of Otorhinolaryngology, Sahlgrenska University Hospital, 41345 Gothenburg, Sweden; gunhildur.gudnadottir@vgregion.se

**Keywords:** feasibility study, tonsil surgery, children, throat pain, recovery, intervention, telephone counseling

## Abstract

Background: Even though children after tonsil surgery experience pain and other limitations in their daily lives, nursing care is transferred to parents after tonsil surgery, and they might need some kind of support. The aim of the study was to test the design of a randomized controlled trial intended to evaluate a nurse-led telephone follow-up after tonsil surgery on postoperative symptoms and quality of life. Methods: Of the seventeen children aged 3–17 years scheduled to tonsil surgery, nine were randomized to the intervention group and eight to the control group using a randomization list. The parents in the intervention group were contacted by telephone on days 1, 3, 5, and 10 postoperatively for counseling by a nurse. The instruments Postoperative Recovery in Children (PRiC) and the health-related quality of life instrument (EQ-5 D-Y) were used to evaluate postoperative symptoms and quality of life, respectively. Results: Eight participants in the intervention group reported throat pain compared to five participants in the control group on the operation day and four days after, possibly due to an uneven distribution of the type of surgery between the study groups. The parents appreciated the telephone counseling, and there were no unplanned revisits in the intervention group. However, it was difficult to recruit participants and the assessment tools were not always fully completed. Conclusions: No explicit conclusions can be drawn from this feasibility study due to the low number of participants and the study design needs adjustments.

## 1. Introduction

Tonsil surgery is one of the most common surgical procedures in the pediatric population. Patients who have undergone tonsil surgery often experience pain and have significant limitations in their daily lives [1,2]. More knowledge about effective interventions that may facilitate parents’ care of their children after tonsil surgery is needed. This feasibility study evaluates a nurse-led telephone follow-up after tonsil surgery concerning postoperative pain, other symptoms, and quality of life among children and adolescents.

In Sweden, 13,000 tonsil surgeries are performed annually as either tonsillectomy or tonsillotomy, often as a day surgery [3]. Common indications for tonsil surgeries are sleep-disordered breathing due to adenotonsillar hypertrophy and recurring tonsillitis. The surgical long-term effects on symptoms and quality of life are beneficial [4]. However, during the immediate postoperative phase, the child or adolescent is likely to suffer from pain [1]. Approximately 4% of the patients are afflicted with postoperative hemorrhage [5]. The highest risk of readmission due to hemorrhage is from postoperative day (POD) 5 to 7 [6]. 

When children undergo tonsil surgery on an outpatient basis, the parents are responsible for managing their postoperative care. To be able to provide their children appropriate care, the parents need competence to adequately manage the children’s postoperative pain and knowledge regarding possible complications. Managing postoperative pain may be particularly demanding when the child is very young [2]. Furthermore, the postoperative care may cause a loss of income for the parent who is absent from work [7]. To improve the caregiver’s confidence and ability to provide adequate postoperative care, several interventions have been developed, such as web-based information [3] or a postoperative follow-up call from a nurse [8]. Despite good intentions by healthcare personnel to facilitate postoperative care, parents nonetheless experience difficulties at home [7]. Paquette et al. [8] showed that children who were provided telephone counseling on POD 1, 3, 5, and 7 increased their intake of fluid and the parents were more likely to administer analgesics on POD 1 and 3. However, there was no significant decrease in the pain intensity reported. Another study found that the parents’ experiences of coping with their child’s pain were influenced by a lack of information, among other factors [2]. 

For the patient and the caregiver, a swift recovery, enhanced well-being, fewer unplanned healthcare contacts, and reduced loss of income are desirable. There seems to be a lack of knowledge about effective interventions that may facilitate the parents’ care of their children after tonsillectomy. Because of that reason, further research is needed, and furthermore, supplementary knowledge is particularly necessary as day surgery is in constant progress, including larger groups and frailer patients [9]. 

The aim of this feasibility study was to assess both the design of and the possibility to conduct a randomized controlled trial intended to evaluate a nurse-led telephone follow-up after tonsil surgery concerning postoperative pain, other symptoms, and quality of life among children and adolescents.

## 2. Materials and Methods

### 2.1. Design 

This study tests the design of and the feasibility to conduct a randomized controlled trial of a nurse-led telephone follow-up, as an additional intervention to usual care, after tonsil surgery in children and adolescents. Data were collected via electronic surveys. This study represents a step in a complex intervention study, as described by the Medical Research Council Guidance (MRC) [10]. 

### 2.2. Settings and Study Population

The participants were recruited from the ear, nose, and throat (ENT) clinics at one tertiary university hospital and one smaller regional hospital in Sweden. All parents of children aged 0–17 years, scheduled to undergo tonsillectomy or tonsillotomy between December 2018 and June 2019 due to recurrent infections or enlarged tonsils, were asked to participate. The parents were informed about the study either by regular staff when visiting the ENT clinics or by letter from E.O. Prior to inclusion, the parents of all children as well as children themselves over the age of 15 years provided written informed consent. Children under the age of 15 years were informed and asked for oral consent. The information emphasized that participation was voluntary and could be terminated at any time. Respondents who had not received information when visiting the clinic were sent a letter with information and a request for participation. A telephone reminder of the request to participate in the study was undertaken by E.O. a few weeks before the planned surgery. Respondents who did not understand Swedish were excluded as well as children with a preoperative disease or condition risking additional complications other than those normally seen after tonsillectomy or tonsillotomy. After having signed the informed consent, the included children were randomized by H.R. using a randomization list according to Polit and Beck (2004). The information according to which the respondents were included in the intervention group was then communicated to E.O., who conducted the telephone counseling. We aimed for a total of 30 participants [11] to be able to carry out the statistical calculations of the result. However, after seven months, the study was closed due to difficulties in recruiting patients. The clinics were repeatedly reminded about the need of more participants. 

### 2.3. Usual Care

Usual care included verbal information by the physician during the presurgical visit to the ENT clinic, a brochure with additional information about the indications for surgery, how to prepare oneself prior to surgery, how the surgery would be performed, risks, postoperative care, and where to address questions. The clinics also referred to the website of The National Tonsil Surgery Register in Sweden (http://www.tonsilloperation.se/en/ accessed on 20 September 2020) [3], for additional information and to watch an animated film where one can follow a child through the tonsillotomy process. The film was produced by the day surgery clinic at the smaller hospital. Children undergoing tonsillectomy at this smaller hospital remained admitted one night after surgery on the pediatric ward or the adults’ ward if over 16 years of age. The younger children met the ENT physician, the anesthesiologist, and the staff one week before surgery. On the adults’ ward, a nurse usually followed up the adolescents undergoing tonsillectomy with a telephone call one week after the surgery. At the larger hospital, children had their surgery at the day surgery clinic without any follow-up.

### 2.4. Intervention

The children and their parents in the intervention group received usual care and prearranged and standardized additional telephone counseling. The child/adolescent or the parent was contacted by telephone on POD 1, 3, 5, and 10 for counseling, support, advice, and referral for a return visit or readmission when needed by E.O., a registered nurse specialized in pediatric care. The manual for the telephone follow-up contained questions about pain; medication; liquid and food intake; complications such as infection, bleeding, severe pain, dehydration, and constipation; and unplanned revisits to the hospital. The telephone follow-up nurse also provided evidence-based advice to the parents to meet each individual child’s needs. The respondents were asked when the child returned to school/preschool and the parents to work.

### 2.5. Instrument and Data Collection

Postoperative Recovery in Children (PRiC) [12] is a questionnaire with 23 questions developed to measure the child’s own experience of their postoperative recovery (see the questions in Table 1). PRiC was tested on Swedish children (*n* = 238) aged 4–12 years who had undergone a tonsil operation in day surgery [12]. PRiC is available in a text-based version in Swedish and English and a version illustrated with photographs. 

The text version of the PRiC instrument was distributed via the web application Research Electronic Data Capture (REDCap) [13] to the parents on the day of surgery and POD 1–7, 10, 14, and 21 to measure symptoms, signs, and well-being. The photo version of PRiC was sent separately upon receiving the signed informed consent to clarify the meaning of the questions in the text version of PRiC. 

The EuroQol-5 Dimension Youth instrument (EQ-5D-Y) (Table 2) is a child-friendly version of the health-related quality of life instrument EQ-5D, tested in several countries and populations [14]. EQ-5D-Y was used 14 days prior to surgery, on the day of surgery, and on POD 2, 10, 14, and 21.

PRiC and EQ5D-Y were distributed to parents and/or the children in both groups. Participants in the intervention group also answered a survey about their satisfaction with the telephone counseling on a five-point Likert scale: “not at all content, not content, content, very content, and extremely content”.

Medical records were used to collect data from participants in both groups concerning unplanned visits postoperatively and demographic data. 

### 2.6. Outcome Measures

The primary outcome was throat pain measured with PRiC [12]. Secondary outcomes were nausea, vomiting, and other symptoms included in the PRiC and/or EQ-5-DY [14]. 

### 2.7. Data Analysis

Quantitative data were analyzed descriptively using IBM SPSS Statistics v.20. No statistical calculations were performed due to the low number of participants.

## 3. Results

**Figure 1:**
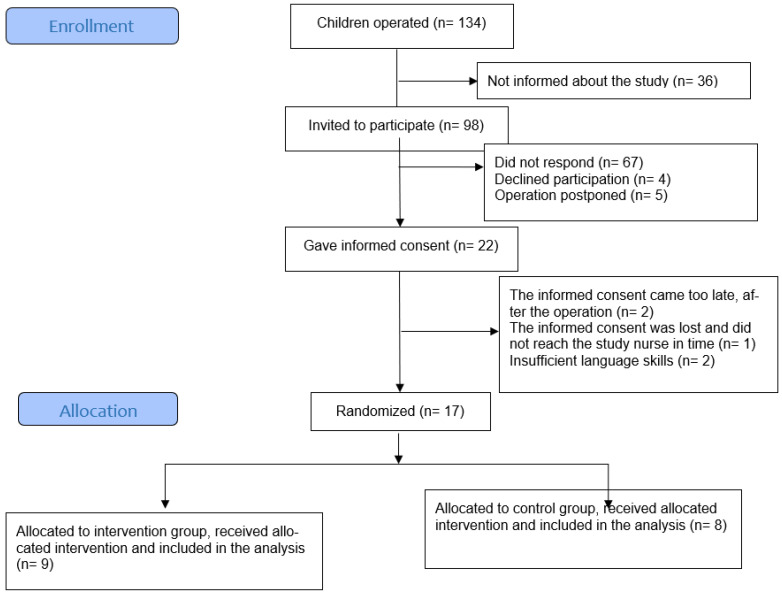
Flowchart of the study.

Of the 98 families invited to the study, 22 participants provided informed consent. Seventeen children and adolescents were included and randomized (Figure 1, flowchart). 

The background data of the participating children (*n* = 17) and children who did not respond (*n* = 5) are described in Table 3. All participants were older than 3 years. The type of surgery was unevenly distributed in the study groups, with five tonsillectomies and four tonsillotomies in the intervention group compared to three tonsillectomies and five tonsillotomies in the control group.

### 3.1. Postoperative Recovery

Most symptoms were reported in both groups during the first six days after surgery, described in Table 4. The symptoms most often reported were difficulties going to preschool/school; throat pain; difficulties playing, eating, and brushing teeth; nausea; earache; difficulties washing/showering; feeling sad; and difficulties talking. After the first week, only a few and mild symptoms were reported in both groups. None reported blood in the mouth, feeling cold, having nightmares, or difficulties urinating on the 21st day after surgery. More participants in the intervention group reported throat pain compared to participants in the control group on the day of surgery, where six participants in the intervention group reported “much” or “very much” pain, compared to one participant in the control group. Postoperatively, the participants in the intervention group reported more pain or the same level of pain compared to the control group on all days except on POD 2. After the first week, only a few and mild symptoms were reported in both groups. None reported blood in the mouth, feeling cold, having nightmares, or difficulties urinating on POD 21.

### 3.2. Quality of Life and Health

EQ-5D-Y showed that the participating children and adolescents had difficulties in engaging in their usual activities and taking care of themselves during the day of surgery, but with fewer difficulties in the following days (Table 5).

### 3.3. Need of Non-Scheduled Care, Postoperative Complications, and Return to School/Preschool

According to telephone follow-up calls on POD 1, 3, 5, and 10, none of the participants who underwent tonsillotomy had contacted any healthcare service. However, one participant who had undergone tonsillectomy in the intervention group booked an emergency visit to the ENT clinic on two occasions (POD 3 and 4), but both visits were cancelled as the symptoms had declined. Three participants in the control group who underwent tonsillectomy contacted the healthcare service, resulting in two telephone calls and five non-scheduled visits at the emergency department. One of these five visits resulted in admittance due to dehydration, pain, and vomiting. None of the nine participants in the intervention group were able to return to school/preschool before POD 5 and four of them were not able to return to school/preschool before POD 10. (Questions about return to school were not posed to the control group.) 

### 3.4. Satisfaction with the Telephone Follow-Up

Only one adolescent (17 years old) answered the questions during the telephone counseling herself. The other respondents were parents, with the children contributing very little or not at all. The participants were satisfied with the counseling (Table 6).

### 3.5. Adverse Events

No adverse events were reported from the intervention.

## 4. Discussion

This feasibility study tested the design of a randomized controlled trial. The present study showed that all parents appreciated the nurse follow-up, rating their satisfaction as “content”, “very content”, or “extremely content”. Furthermore, no unplanned revisits in the IG occurred (a finding that could be compared to five unplanned visits in the control group), suggesting that the nurse-led follow up can be a valuable intervention to support parents and prevent unnecessary hospital revisits. These findings have to be confirmed in future studies. An earlier study [15] with 863 participants found that nurse-led telephone follow-up on POD 1, 3, 7, and 14 reduced healthcare seeking when compared to visits to a physician on the same postoperative days. On the other hand, Paquette et al. [8] did not find any significant differences regarding the use of healthcare resources in a study that compared nurse-led telephone follow-up on POD 1, 3, 5, and 10 to children who only received standard care with no follow-up, which may be due to the low number of participants (*n* = 45).

Although both PRiC and EQ-5D-Y are considered to be valid and reliable instruments, several similar questions are found in both that may have been confusing for the respondents. EQ-5D-Y was additionally used to illustrate the child’s perceived quality of life. This instrument has been proved to be satisfactorily understood by children over 8 years in different countries [14]. A limitation is that we used the instrument also for children under the age of 8 years. The use of PRiC, designed to provide the newly operated child a voice, was valuable. For future studies, we recommend using only PRiC.

The manual for the telephone intervention was perceived as helpful for the telephone-counselling nurse in mapping the current situation and the child’s needs. Therefore, the manual is recommended for future studies. A similar document could also help nurses at ENT clinics to provide advice to parents about appropriate care during their child’s postoperative recovery.

Although statistical analysis could not be performed due to few participants, perceived symptoms in both groups have been reported, providing an understanding of the severity of postoperative complaints. Previous research has shown that children who have undergone tonsillectomy experience more pain than those who have undergone tonsillotomy [16]. This fact could explain the result that the children in the intervention group seemed to experience more pain, since there were more tonsillectomies in the intervention group compared to the control group. An additional hypothesis is that the children in the intervention group reported more pain because of the extra attention they received by obtaining the telephone counseling [17]. This extra attention might lead not only to more reported pain but also to increased pain medication, thereby being beneficial for the child.

Both the intensity and the cause of the preoperative pain are important to examine. EQ5D-Y showed that 11 of the 17 children in the study experienced pain preoperatively, which could have affected their experience of pain postoperatively. In addition, in future studies, the documentation of the consumption of analgesics is needed to possibly distinguish the effect of drugs from telephone counseling.

PRiC has previously been shown to be a valuable instrument to follow the impact of interventions after tonsil surgery in children [16]. When used in the present study, the symptoms most often reported turned out to be the same as those in the study by Alm et al. [16], in which postoperative symptoms were examined with PRiC in 299 children on POD 3.

A limitation was that only 17 participants undergoing either tonsillectomy or tonsillotomy were recruited. In spite of many parents being initially interested in participating, few parents returned the written consent by mail. For a future randomized controlled trial, we recommend recruitment through the digital handling of the informed consent via a QR code. There is a need of at least 12 participants per group for statistical analysis [11]; therefore, this was not performed and differences between the groups cannot be confirmed. 

Another limitation was the non-completion of the questionnaires, known to be a common problem in surveys [11]. In our study, the use of two instruments with several similar questions, sent out on eleven occasions, could have made participants less motivated to answer. With the large age range of 3–17 years, the developmental stages of the participants as well as the parents’ need for support would probably vary greatly, and some questions did not suit all children. We note the importance of careful planning for successful recruitment and choice of instruments and frequency of data collection [18]. For future studies, we recommend age-specific instruments, sent out on fewer occasions, to facilitate data collection.

Also, the children included in the study underwent tonsillectomy or tonsillotomy, and we know that the pain level and speed of recovery vary considerably depending on type of surgery [1]. This is also a confounding factor when examining the effect of telephone counseling, and the outcome analysis should therefore be conducted separately, one for each type of surgery.

To find out whether telephone follow-up is a valuable and feasible part of the postoperative routine for children who have undergone tonsil surgery, interviews with staff and children need to be conducted to understand their experiences.

## 5. Conclusions

A nurse-led manual-based telephone support after tonsil surgery in children and adolescents is probably a helpful support for parents and children to manage postoperative pain and other symptoms, and may reduce unplanned and unnecessary revisits to the hospital. The preliminary results show that the participants who received telephone counseling from a nurse were satisfied with this support. In this feasibility study, we identified some possible improvements in the study design that we plan to implement in a future randomized controlled study. 

## Figures and Tables

**Table 1:** Questions in the PRiC instrument.

During the Last Day/Night–within the Last 24 h		Not at All	A Little	Much	Very Much
1	Have I felt like vomiting/throwing up?				
2	Have I vomited/thrown up?				
3	Have I been feeling cold?				
4	Have I been dizzy?				
5	Have I had a sore throat?				
6	Have I had a stomach ache				
7	Have I had an earache?				
8	Have I had an headache				
9	Have I felt sad?				
10	Have I had frightening dreams?				
11	Have I had difficulty urinating				
12	Have I had difficulty defecating?				
13	Have I had blood in my mouth?				
14	Have I had difficulty breathing?				
15	Have I had difficulty sleeping?				
16	Have I had difficulty eating?				
17	Have I had difficulty playing/being active?				
18	Have I had difficulty resting?				
19	Have I had difficulty talking?				
20	Have I had difficulty brushing my teeth?				
21	Have I had difficulty washing myself/showering?				
22	Have I had difficulty attending daycare/school?				
		**Very well**	**Pretty well**	**Pretty bad**	**Very bad**
23	At the moment I feel				

Mark the box that best describes how you have felt since your surgery.

**Table 2:** Questions in the EQ-5D-Y instrument.

Variables	Grade
Mobility (walking about)	No problems, some problems, a lot of problems
Looking after myself	No problems, some problems, a lot of problems
Doing usual activities (for example, going to school, hobbies, sports…)	No problems, some problems, a lot of problems
Having pain or discomfort	No pain, some pain, a lot of pain
Feeling worried, sad or unhappy	Not worried, a bit worried, very worried

**Table 3:** Demographics and baseline characteristics (*n* = 17).

	Intervention Group (*n* = 9)	Control Group (*n* = 8)	Eligible but Not Participating (*n* = 81)
Gender			
Male	6	6	41
Female	3	2	33
**Age**			
<3 years			6
3–7 years	6	6	57
8–12 years	2	1	8
13–17 years	1	1	3
**Type of surgery**			
Tonsillectomy	5	3	
Tonsillotomy	4	5	
**Main indication for surgery**			
Tonsillar hypertrophy	6	6	
Recurrent tonsillitis	3	2	
**Length of hospital stay**			
<24 h (day surgery)	7	7	
>24 h (one night’s observation)	2	1	
**Hospital**			
Larger	6	4	
Smaller	3	4	

**Table 4:** Throat pain (the primary outcome), difficulty in playing, difficulty in eating, nausea, difficulty in brushing teeth, and earache on operation day (OD) and postoperative day (POD) 2, 4, and 6 for the intervention group (IG) and control group (CG), as reported in PRiC.

	OD		POD 2		POD 4		POD 6	
Intervention Group (IG) Control Group (CG)	IG (*n* = 9)	CG (*n* = 8)	IG (*n* = 9)	CG (*n* = 8)	IG (*n* = 9)	CG (*n* = 8)	IG (*n* = 9)	CG (*n* = 8)
**Throat pain**								
Not at all	0	0	0	0	1	1	2	1
A little	2	4	3	4	4	3	2	2
Much	3	0	1	2	4	1	2	1
Very much	3	1	1	1	0	1	1	1
Missing values	1	3	4	1	0	3	2	3
**Difficulty in playing**								
Not at all	0	1	2	3	2	2	1	1
A little	4	3	1	2	5	1	2	3
Much	1	0	2	0	2	0	3	0
Very much	3	1	0	2	0	2	1	1
Missing values	1	3	4	1	0	3	2	3
**Difficulty in eating**								
Not at all	0	1	2	3	2	2	1	1
A little	4	3	1	2	5	1	2	3
Much	1	0	2	0	2	0	3	0
Very much	3	1	0	2	0	2	1	1
Missing values	1	3	4	1	0	3	2	3
**Nausea**								
Not at all	2	4	3	5	6	5	5	4
A little	4	0	2	1	3	1	1	0
Much	1	1	0	1	0	0	0	0
Very much	1	0	0	0	0	0	1	1
Missing values	1	3	4	1	0	3	2	3
**Difficulty in brushing teeth**								
Not at all	5	4	5	7	9	6	6	5
A little	2	0	0	0	0	0	0	0
Much	1	1	0	0	0	0	0	0
Very much	0	0	0	0	0	0	1	0
Missing values	1	3	4	1	0	3	2	3
**Earache**								
Not at all	7	5	2	7	5	3	4	3
A little	1	0	2	0	2	1	1	1
Much	0	0	1	0	1	1	1	0
Very much	0	0	0	0	1	1	1	1
Missing values	1	3	4	1	0	3	2	3

The bold text in the left column represents question heading. No complications led to unplanned visits postoperatively in the intervention group, while three unplanned visits due to postoperative complications occurred in the control group.

**Table 5:** EQ5DY showing perioperative difficulties in engaging in usual activities.

EQ5DY	PreOP		OD		POD10		POD14		POD21	
Intervention Group, IG (*n* = 9) Control Group, CG (*n* = 8)	IG	CG	IG	CG	IG	CG	IG	CG	IG	CG
Q1 Mobility (walking about)										
no problems	9	8	5	5	6	5	5	4	6	5
some problems	0	0	2	1	0	0	0	0	0	0
a lot of problems	0	0	1	0	0	0	0	0	0	0
Missing values	0	0	1	2	3	3	4	4	3	3
Q2 Looking after myself *										
no problems	8	5	4	2	6	4	5	4	6	4
some problems	0	0	2	1	0	0	0	0	0	0
a lot of problems	0	0	1	0	0	0	0	0	0	0
Missing values	1	3	2	5	3	4	4	4	3	4
Q3 Doing usual activities **										
no problems	8	8	0	1	4	4	6	4	5	4
some problems	1	0	3	4	1	0	0	1	1	1
a lot of problems	0	0	5	1	0	0	0	0	0	0
Missing values	0	0	1	2	4	4	3	3	3	3
Q4 Having pain or discomfort										
no pain	4	2	0	0	4	3	4	3	6	4
some pain	5	5	5	4	2	2	1	1	0	1
a lot of pain	0	1	3	2	0	0	0	0	0	0
Missing values	0	0	1	4	3	3	4	4	3	3
Q5 Feeling worried, sad, or unhappy										
not	5	7	2	2	6	4	5	4	6	5
a bit	4	1	5	4	0	1	0	0	0	0
very	0	0	1	0	0	0	0	0	0	0
Missing values	0	0	1	2	3	3	4	4	3	3
Q6, How good is your health TODAY? 0–100, median	81	86	49	71	92	85	97	85	96	94

Additional information to participants: * Skip this issue if the child has not yet learned to take care of himself. ** For example, going to school, sports and leisure activities, playing, and doing things with family or friends. Abbreviations: PreOP = Preoperation; OD = Operation day; POD = Postoperative day.

**Table 6:** Satisfaction with the telephone follow-up.

How Satisfied Are You with the Telephone Counseling You Received? Choose the Option That Best Matches How Satisfied You Are.					
	POD * 1	POD 3	POD 5	POD 10	Total
Not at all content	0	0	0	0	0
Not content	0	0	0	0	0
Content	2	2	2	2	8
Very content	4	3	3	3	13
Extremely content	0	1	2	1	4
Total	6	6	7	6	25

* POD = postoperative day.

## Data Availability

The data has been deposited into a publicly available repository and can be obtained on request.
